# Health Risk Assessment of Potentially Toxic Elements Contamination of Commonly Consumed Fruits in Bahir Dar Town, Northwest Ethiopia

**DOI:** 10.1155/2024/6677324

**Published:** 2024-05-15

**Authors:** Biset Asrade Mekonnen, Muluabay Getie Yizengaw, Haile Kassahun, Gebremariam Ketema

**Affiliations:** ^1^Department of Pharmacy, College of Medicine and Health Sciences, Bahir Dar University, Bahir Dar, P.O. Box 79, Ethiopia; ^2^Department of Biochemistry, College of Medicine and Health Sciences, Bahir Dar University, Bahir Dar, P.O. Box 79, Ethiopia; ^3^Department of Pharmacy, College of Medicine and Health Sciences, Wollo University, Dessie, Ethiopia

## Abstract

**Background:**

Excess accumulation of potentially toxic elements in frequently consumed fruits is a serious threat to human health. The aim of this study was to determine the levels of cadmium (Cd), chromium (Cr), copper (Cu), and lead (Pb) and to estimate the noncarcinogenic and carcinogenic health risks associated with their daily intake in commonly consumed fruits in Bahir Dar town, northwest Ethiopia.

**Methods:**

Three types of fruits (mango, banana, and orange) were collected using a simple random sampling method from open markets in Bahir Dar town. Wet digestion was used, and the concentration of potentially toxic elements was analyzed in the edible portion of the fruits. The average daily intake (ADI), hazard quotient (HQ), hazard index (HI), and target cancer risk (TCR) were estimated to assess the human health risks posed by the intake of those potentially toxic elements from the consumption of the studied fruits.

**Results:**

The concentrations of Cr and Cu were lower than the maximum limit of normal values in mango, banana, and orange. However, the Pb and Cd concentrations (mg kg^−1^) in mango were 0.576 and 1.771, respectively, which exceeded the FAO/WHO recommended permissible limits of 0.3 mg kg^−1^ and 0.2 mg kg^−1^, respectively. The ADI of potentially toxic elements was found to be lower than the maximum permitted tolerable daily intake in the studied fruits, and the HI values (mgday^−1^ kg^−1^) of all studied potentially toxic elements were lower than one in banana and orange, except mango (3.69). The TCR values for Pb, Cd, and Cr in banana and orange were 7.16 × 10^−4^ and 7.15 × 10^−4^, respectively, which exceeded the recommended threshold risk limit (>1 × 10^−4^), but in mango (1.71 × 10^−3^), the level was above the moderate risk limit (>1 × 10^−3^). However, the TCR value in all the studied fruits was above the recommended safe limit (ILCR < 1 × 10^−6^) set by the United States Environmental Protection Agency (US-EPA).

**Conclusion:**

The study revealed that the consumption of mango fruit in the studied areas may pose noncarcinogenic and carcinogenic adverse health effects.

## 1. Introduction

Fruits are an important component of the human diet because they are a source of essential micronutrients such as copper (Cu), zinc (Zn), calcium (Ca), iron (Fe), magnesium (Mg), iodine (I), sodium (Na), potassium (K), vitamins, and fibers and have beneficial antioxidative effects [1]. The intake of various fruits has become the main source of nutrients, which are an important part of the human diet required for the maintenance of health, prevention, and treatment of various communicable and noncommunicable diseases [2, 3].

Fruits have recently attracted great interest as potential therapeutic agents against a variety of diseases like those involving radical damage due to the presence of lipotropic, antioxidant, and antitumour properties which have various activities such as antimicrobial, anti-inflammatory, antimutagenic, and antioxidative activities. These activities are essential to boost immunity, strengthen bones, lower cholesterol levels, prevent anemia, alleviate symptoms associated with gastrointestinal disorders (gastritis, peptic and duodenal ulcers, and irritable bowel syndrome), improve digestive health, and support eye, hair, skin, and heart health. They also show beneficial effects in age-related cardiovascular diseases, some forms of cancer, and Alzheimer's diseases [3–5].

However, the contamination of fruits with contaminants from the soil and atmosphere questions their quality and safety [6]. Potentially toxic elements are among the major contaminants in fruits and may be considered a major problem in our environment. Such a problem is becoming more serious worldwide, especially in developing countries [1, 3, 6].

Potentially toxic elements are not biodegradable, have long biological half-lives, and can accumulate in different body organs, leading to unwanted side effects [3]. They have drawn much attention because of their ubiquity, trace levels of toxicity, and persistence in the environment. The most commonly found potentially toxic elements include cadmium (Cd), chromium (Cr), copper (Cu), and lead (Pb), all of which pose risks to human health even at trace concentrations worldwide, mainly in developing countries, including Ethiopia [3, 7–10].

Currently, in Ethiopia, there is no regulatory criterion for potentially toxic elements in irrigation water, soils, and fruits. However, due to industrial and agricultural development, there is concern over the adverse effects of inorganic fertilizers, pesticides, animal manure, and mining activities, which affect the soil and water supply. These agrochemicals leave residues such as potentially toxic elements that pose health risks to humans and hazardous ecological risks to plants, animals, and microorganisms [3, 10].

In Ethiopia, fruits are the most widely consumed, produced, and exported staple food. If they are contaminated by potentially toxic elements, they can put the population at risk [7, 10]. Banana (*Musa Cavendish L.*), orange (*Citrus aurantium L.*), and mango (*Mangifera indica L*.) are the most widely produced, consumed, and exported fruit crops in Ethiopia [10]. However, the overall consumption, production, and exportation of these fruits are quite small because of the low volume of production, high costs of production, and poor quality of production [10].

Therefore, this study is designed to explore (i) the degree of potentially toxic element contamination level in the selected fruits, (ii) public health risks associated with the consumption of these fruits contaminated with potentially toxic elements by measuring the average daily intake (ADI) of potentially toxic elements earlier identified from the study area, and (iii) predict the potential cancerous and noncancerous health risks of the population in the study area by integrating all the information at quantitative estimates of target cancer risk (TCR) and hazard index (HI) of chromium (Cr), cadmium (Cd), lead (Pb), and copper (Cu) associated with these potentially toxic elements in the selected fruits [11]. The study is intended to benefit the population and concerned bodies as they may appreciate the benefit of adopting various fruit quality and safety control practices that may enable them to improve the quality and safety of fruit production. The results will also provide invaluable baseline data for further investigation of potentially toxic element accumulation in fruits, thereby improving food safety and the health of its inhabitants.

## 2. Materials and Methods

### 2.1. Equipment, Instruments, and Apparatus

Flame atomic absorption spectrophotometer (Buck Scientific Model 210VGP AAS, USA), ceramic mortar with a pestle (Halden Wanger, Germany), digital analytical balance (Mettler Toledo, E11140, Switzerland), hot plate (Stuart Scientific, UK), Whatman No. 42 filter paper (Whatman Limited, England), polyethylene bags, measuring cylinders, beaker, pipette, volumetric flasks, conical flask, biological safety cabinet, refrigerator, funnel, porcelain crucibles, hot air oven, and plastic knife were used.

### 2.2. Chemicals, Regents, and Solvents

Analytical graded chemicals, reagents, and solvents were used throughout the experiment. Deionized water, HClO_4_ (70%, Sisco Pvt Ltd., India), HNO_3_ (69%, Oxford Lab. Chem., India), H_2_O_2_ (30%, Scharlab S. L., Spain), Cd (NO_3_)_2_, Pb (NO_3_)_2_, Cu (NO_3_)_2_, and Cr (NO_3_)_2_ (99.99%, Merck, Germany) were used.

### 2.3. Study Area and Period

**Figure 1:**
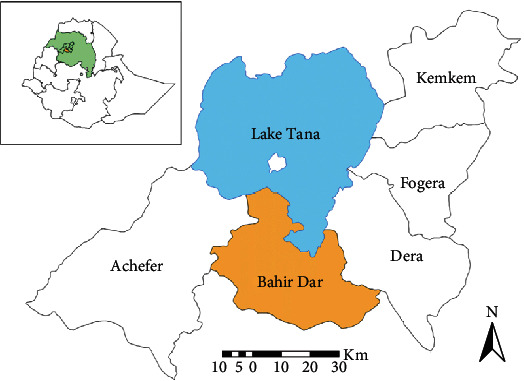
Location map of the study area in Northwest Ethiopia.

The study was conducted in Bahir Dar town, Northwest Ethiopia, from January 2021 to June 2021. Bahir Dar, the capital city of Amhara National Regional State, is 552 km away from Addis Ababa, the capital city of Ethiopia, and is located at 11036′ N, 37023′ E on the southern shore of Lake Tana, where the Blue Nile River starts (Figure 1). It is a rapidly expanding town with commercial centers, small industries, and residences in all sectors [10, 12].

### 2.4. Study Design and Sample Collection Methods

An experimental-based study design was used. Highly consumable fruits such as mango, banana, and orange in the study area were selected [10]. One hundred twenty fresh fruit samples (*n* = 40, for each) were collected from 20 randomly selected retailers and vendors at the Bahir Dar town open market during the study period. The collected samples were then mixed to form a composite sample (5 kg), and 1.25 kg of the composite sample was used as a subsample [12, 13].

### 2.5. Sample Preparation and Treatment

The collected fresh fruit samples were washed thoroughly with clean tap water and then washed three times with deionized water. The cleaned samples were peeled to separate the edible parts from the nonedible parts using a clean knife. The edible portions were sliced and dried in an oven at 105°C for 24 h. After drying, the samples were homogenized by grinding with a glass mortar and pestle and then sieved through a 2 mm nylon sieve to remove coarse debris. The fine and homogenized powder samples were stored in plastic-sealed bags with proper labels until they were used for wet digestion [12–15].

### 2.6. Digestion and Analysis of the Samples

One gram of the oven-dried and homogenized sample was measured and added into a 200 mL conical flask, followed by the addition of a triacid mixture of 10 mL HNO_3_ (69%), 4.0 mL HClO_4_ (70%), and 4.0 mL H_2_O_2_ (30%). The mixture was then digested at 240°C for 2 h on a hot plate and in a biological safety cabinet until a clear and colorless solution was obtained. After digestion was completed, the solution was cooled, filtered through Whatman No. 42 filter paper using a 50 mL volumetric flask, and finally diluted with deionized water to the mark [12, 16, 17].

A blank solution containing only a mixture of 10 mL HNO_3_ (69%), 4.0 mL HClO_4_ (70%), and 4.0 mL H_2_O_2_ (30%) was prepared using the same procedures used for the sample preparations. All digested and blank solutions were stored in a refrigerator at 4°C until analysis using a flame atomic absorption spectrophotometer (FAAS) (Buck Scientific Model 210VGP AAS, USA) at the Department of Chemistry, University of Gondar. Triplicate digestion and analysis were performed, and the results were reported in mg/kg dry weight of the sample [12, 18].

### 2.7. Calibration Curve Procedure

Standard solutions of 1000 mg/L of Pb (NO_3_)_2_, Cd (NO_3_)_2_, Cr (NO_3_)_2_, and Cu (NO_3_)_2_ were diluted to obtain an intermediate standard solution of 10 mg/L using a 50 mL volumetric flask and finally diluted with deionized water to the mark. A five-point calibration curve was constructed using five series of working standard solutions prepared by serial dilution with deionized water from the respective intermediate standard solutions. The concentration and measured absorbance values of each potentially toxic element with their respective wavelengths were plotted after calibrating the FAAS instrument (Table 1) [12, 13, 19–21].

### 2.8. Method Validation

Method detection limit, quantification limit, precision, and accuracy tests were conducted to validate the analytical method and the efficiency of the FAAS instrument [16, 17].

#### 2.8.1. Accuracy

The accuracy of the method was assessed by spiking preanalyzed samples of 1 g of fruit with known amounts of standard potentially toxic elements (0.5 ppm), and the percentage recovery was calculated to evaluate the accuracy of the analytical procedure using the following equation [16, 17]:

(1) $$\begin{array}{c} \text{Recovery }\left(\%\right)=\frac{\text{CM in the spiked sample}\left(\text{mg}/\text{L}\right)-\text{CM in the unspiked sample}\left(\text{mg}/\text{L}\right)}{\text{CM added for spiking }\left(\text{mg}/\text{L}\right)}\times 100, \end{array}$$

where CM is the concentration of potentially toxic elements.

#### 2.8.2. Precision

The repeatability of the analytical procedure was checked by performing a triplicate analysis from triplicate digested samples (*n* = 9) using Eq. (2). The obtained results as the average of three replicates of each sample showed the validity of the employed methods and good repeatability for the analysis of fruit samples [16, 17].

(2) $$\begin{array}{c} \text{RSD }\left(\%\right)=\frac{\text{Standard deviation}}{\text{Mean value}}\times 100. \end{array}$$

#### 2.8.3. Method Detection and Quantification Limits

The method detection limit (MDL) is the concentration that gives a signal three times the standard deviation of the blank or background signal, whereas the method quantification limit (LOQ) is the concentration that gives a signal ten times the standard deviation of the blank or background signal. The standard deviation for each potentially toxic element was calculated from the nine blank measurements to determine MDL and LOQ for each element using the following equations [12] [16, 17]:

(3) $$\begin{array}{c} \text{MDL}=3\times S\text{ of the blank}, \end{array}$$

(4) $$\begin{array}{c} \text{LOQ}=10\times S\text{ of the blank}, \end{array}$$

where *S* is the standard deviation of the nine blank measurements.

### 2.9. Determinations of Potentially Toxic Elements

The mean concentration value of each element was determined in dry weight using the following equation [11, 19]:

(5) $$\begin{array}{c} \text{Conc}.\text{in dry weight }\left({\text{mgkg}}^{-1}\right)=\frac{\left(C_{s}-C_{b}\right)\times V\left(L\right)\times \text{CF}}{W}, \end{array}$$

where *C*_*s*_ and *C*_*b*_ are the concentrations of potentially toxic elements in the fruit sample and blank solutions in mgL^−1^, respectively; *V* is the final volume (50 mL) of the digested fruit sample solution in liters; *W* is the initial weight (1 g) of each fruit sample measured in kilograms; and CF is the conversion factor (0.085) [18–20].

### 2.10. Health Risk Assessment of Potentially Toxic Elements

To assess the potential health risks associated with long-term ingestion of potentially toxic element-contaminated fruits, the average daily intake (ADI), hazard index (HI), target hazard quotient (THQ), and target carcinogenic risk (TCR) of potentially toxic elements were used [11, 20].

#### 2.10.1. Average Daily Intake (ADI)

The ADI value depends on the concentration of potentially toxic elements in fruits, the amount of daily consumption, and body weight. The ADI was calculated using the following equation [11, 15]:

(6) $$\begin{array}{c} \text{ADI}=\frac{\left(C_{\text{element}}\times \text{IR}\right)}{\text{BW}}, \end{array}$$

where ADI is the average daily intake of potentially toxic elements (mgkg^−1^day^−1^) in fruits; *C*_element_ is the average concentration of potentially toxic elements in the edible portion of fruits (mgkg^−1^, dry weight), which is determined using Eq. (5); IR (ingestion rate) is the average daily fruit consumption rate for the Ethiopian (adult), which is 115 g person^−1^day^−1^ [10, 22]; and BW is the reference body weight for an adult (70 kg [7, 14, 23].

#### 2.10.2. Hazard Quotient (HQ) and Hazard Index (HI)

The hazard quotient (HQ) is the ratio of the determined dose of a contaminant to the oral reference dose, Eq. (7), which is used to estimate the noncarcinogenic risk of potentially toxic elements contaminated fruits [11, 20].

(7) $$\begin{array}{c} \text{HQ}=\frac{\text{ADI }}{RfD}, \end{array}$$

where ADI is the average daily intake of potentially toxic elements in fruits in mgkg^−1^day^−1^ determined using Eq. (6) and *RfD* is the oral reference dose of the potentially toxic elements (mgkg^−1^day^−1^), which is an estimated exposure of elements to the human population per day that has no hazardous effect during a lifetime and is used in the EPA's noncancer health risk assessments. The values of *RfD* (mgkg^–1^day^−1^) for Pb, Cd, Cr, and Cu are 0.0035, 0.001, 0.003, and 0.04, respectively [11, 20].

The hazard index (HI) has been used to estimate the overall noncarcinogenic risk to human health through exposure to more than one potentially toxic element in the same fruit. If the HI value is greater than 1, it indicates that the population will pose potential adverse health effects. The HI valve was determined using the following equation [3, 11, 20, 23, 24]:

(8) $$\begin{array}{c} \text{HI}=\sum \text{H}\text{Q}=\text{HQpb}+\text{HQCu}+\text{HQCr}+\text{HQCd}, \end{array}$$

where Pb, Cu, Cr, and Cd are the individual potentially toxic elements found in each fruit species.

#### 2.10.3. Target Cancer Risk (TCR)

Target cancer risk (TCR) is used to estimate the carcinogenic risk of potentially toxic element consumption. The TCR values of Pb, Cd, and Cr in the selected fruits were estimated using the following equations [11, 15, 20, 23, 24]:

(9) $$\begin{array}{c} \text{ILCR}=\text{ADI}\times {\text{CSF}}_{\text{o}}, \end{array}$$

(10) $$\begin{array}{c} \text{TCR}=\sum \text{I}\text{LCR}={\text{ILCR}}_{\text{Pb}}+{\text{ILCR}}_{\text{Cr}}+{\text{ILCR}}_{\text{Cd}}, \end{array}$$

where ILCR represents the incremental lifetime cancer risk by individual potentially toxic element ingestion in fruits, ADI is the average daily carcinogenic element intake of the population in mgkg^−1^day^−1^ body weight, and CSF_o_ is the oral cancer slope factor in mgkg^−1^day^−1^, which is the risk produced by the average dose of 1 mgkg^−1^day^−1^ that has values (mgkg^−1^day^−1^) for Pb, Cd, and Cr of 0.0085, 0.38, and 0.5, respectively [11, 20].

### 2.11. Data Analysis

The experimental data were analyzed using SPSS version 23 and ANOVA, and the results are reported as the mean ± SD of triplicate analysis using tables and charts. All statistical tests were conducted at a 95% confidence level. A two-tailed test with *P* values < 0.05 at 95% Cl was declared as a significant difference.

## 3. Results and Discussion

### 3.1. Method Validation

The Pb, Cd, Cr, and Cu recovery test results in the selected fruit samples with matrix spikes in percentage were 96–107, 96–101.6, 90.8–104.2, and 91.5–100.5, respectively, and the %RSD values were 0.03–4.9, 0.03–3.1, 0.04–2.9, and 0.2–7.7, respectively. All recovery and %RSD values were within the acceptable range of 80%–120% and ≤10%, respectively. The instrumental detection limit (IDL) for all metals was Pb (0.082), Cr (0.051), Cd (0.020), and Cu (0.009), below the method detection limit (MDL), which indicated that the analytical method and the FAAS instrument (Buck Scientific Model 210VGP AAS, USA) are precise, accurate, and sensitive for the analysis of the selected potentially toxic elements at trace levels [12, 23, 25].

### 3.2. Concentration of Potentially Toxic Elements in Fruits

The mean concentrations of Pb and Cd in mango were 0.576 mgkg^−1^ and 1.771 mgkg^−1^, respectively, which exceeded the FAO/WHO limit standards [20]. The mean concentrations and range of potentially toxic elements found in the selected fresh fruit samples collected from the local open markets in Bahir Dar, Ethiopia, are summarized in Table 2.

**Figure 2:**
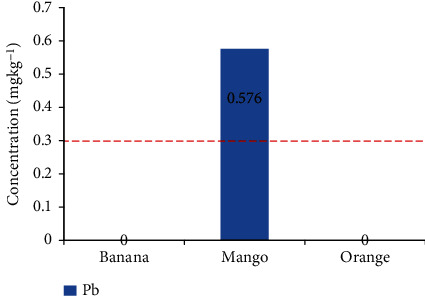
Comparison of lead concentrations in fruits with FAO/WHO standard limits.

Lead (Pb) is a serious cumulative body poison that enters the body system through air, water, and food and cannot be removed by washing fruits [3, 6, 11, 20]. In this study, the highest concentration of Pb was observed in mango (0.576 mg kg^−1^), which exceeded the FAO/WHO stipulated safe limits of 0.3 mgkg^−1^ (Table 2 and Figure 2) [20]. The findings of this study were greater than those of other similar studies conducted in Uganda (0.32 mgkg^−1^) [26] and Addis Ababa (0.25 mgkg^−1^) [27], but less than those of studies conducted in Libya (1.824 mgkg^−1^) [28]. This is because the overuse of fertilizers in soils may increase the quantity of Pb in the soil [12, 20]. This result indicated that mango has an unsafe level of Pb, which may cause central nervous system disorders, acute and chronic kidney disease, gastrointestinal disturbances, slight liver impairment, and damage to the reproductive system of consumers [3, 9, 20].

Cadmium (Cd) is a nonessential element in foods that accumulates principally in the kidney and liver [3, 9, 15]. The lowest and highest concentrations (mgkg^−1^) of Cd were found in banana (0.130) and mango (1.771 mgkg^−1^), respectively. The Cd concentration level in mango exceeded the FAO/WHO stipulated safe limit of 0.2 mgkg^−1^ [20] and the findings of other similar studies conducted in Libya (0.362 mgkg^−1^) [28]. Because of industrial and agricultural development, the extensive use of inorganic fertilizers, pesticides, waste incineration, and mining activities, which harm the soil and groundwater, and the use of untreated groundwater for irrigation purposes may increase the level of Cd in mango [20, 27, 29]. This result revealed that mango has an unsafe level of cadmium, which may accumulate in the kidneys and damage the filtering mechanisms. It also causes diarrhea, stomach pains, severe vomiting, bone fractures, damage to the central nervous system and immune system, reproductive failure, and possibly DNA damage [3, 9, 11, 20]. The overall concentration of Cd (mgkg^−1^) in the edible portions of fruit samples was found to be in mango (1.771) > orange (0.147) > banana (0.130) (Table 2).

Chromium (Cr) concentrations in the edible portions of fruit samples ranged between 0.728 mgkg^−1^ and 0.773 mgkg^−1^. The highest and lowest concentrations of Cr were found in bananas (0.773) and mango (0.728), respectively. The concentration of Cr in all fruit samples was below the FAO/WHO safe limit of 2.3 mgkg^−1^ [20], but higher than the findings of other similar studies conducted in Uganda (0.4 mgkg^−1^) [26]. This result revealed that all the studied fruits have a safe level of Cr, which may not pose health risks to consumers. The overall concentration of Cr (mgkg^−1^) in the edible portion of the fruit samples was found to be in banana (0.773) > orange (0.759) > mango (0.728) (Table 2).

Copper (Cu) is an essential trace element required for proper health within an appropriate limit [3, 11, 20]. High copper uptake in fruits can be harmful to human health, whereas low copper uptake in humans can cause growth retardation, skin ailments, and gastrointestinal disorders [30]. Copper was detected in all types of the selected fruit samples in the range between 0.138 mgkg^−1^ and 2.699 mgkg^−1^. The minimum and maximum concentrations were found in banana (0.138) and mango (2.699), respectively. The findings of this study were within the FAO/WHO safe limit of 40 mgkg^−1^ [20]. The findings of this study are also similar to those of other studies conducted in Addis Ababa [27] and Libya [28]. This result indicates that all the studied fruits have a safe level of Cu, which may not pose health risks to consumers. The overall concentration of Cu (mgkg^−1^) in the edible portions of fruit samples was found to be in mango (2.699) > banana (0.138) > orange (0.138) (Table 2).

### 3.3. Health Risk Assessment of Potentially Toxic Elements

#### 3.3.1. Average Daily Intake (ADI) of Potentially Toxic Elements

There are many pathways for potentially toxic element exposure in humans. Ingestion of fruits contaminated with significant amounts of potentially toxic elements could harm human health [3, 11, 20]. In this study, the ADI values for Pb, Cd, Cr, and Cu were below the FAO/WHO and RfD values in all fruit samples, but the ADI value of Cd was above the RfD value in mango (Table 3) [11].

#### 3.3.2. Hazard Quotient (HQ) and Hazard Index (HI) of Potentially Toxic Elements

**Figure 3:**
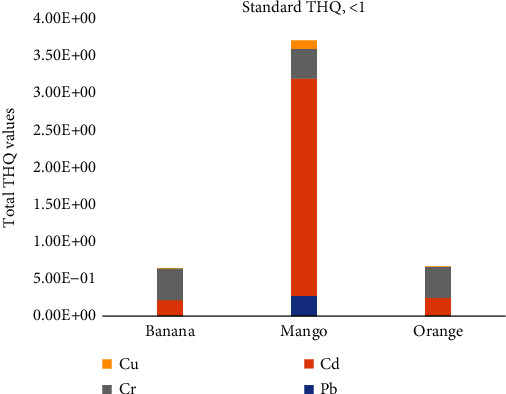
Comparison of the total THQ values of potentially toxic elements in fruits with the US-EPA standard limit.

The HI values of all studied potentially toxic elements were lower than one in each fruit sample except mango (3.69) compared with the US-EPA upper limit (Figure 3) [11]. This indicates that this population may face a noncancer health risk caused by the intake of Cd via the consumption of mango in their lifetime. However, the population does not face a noncancer health risk via the consumption of bananas or oranges in their lifetime [8, 11, 20].

#### 3.3.3. Target Cancer Risk (TCR) of Potentially Toxic Elements

Pb, Cd, and Cr (IV) are classified by the International Agency for Research on Cancer (IARC) as carcinogenic agents [11, 20]. The cumulative incremental lifetime cancer risk (∑ILCR) of all analyzed potentially toxic elements in the studied fruits exceeded the recommended threshold risk limit (>1 × 10^−4^) (Table 4) [11].

However, mango has the highest chance of cancer risks (1.71 × 10^−3^), which was above the moderate risk limit (>1 × 10^−3^). This result indicated that the consumption of mango from the study area would result in an excess of 17 cancer cases per 10,000 people exposed [11]. Among the studied potentially carcinogenic elements, Cd is the major risk contributor, accounting for 64.9% of the potentially carcinogenic elements in the studied fruits. Therefore, Cd was the most dominant carcinogen in the analyzed fruits in the study area. Thus, attention should be paid to controlling its exposure to the environment to save the population from cancer risk [3, 11, 20, 30].

## 4. Conclusions

The results of this experimental study indicated that the concentrations of Cr and Cu in mango, orange, and banana were lower than their respective FAO/WHO limits. However, the concentrations of Pb and Cd in mango were higher than the limit standards. The hazard index (HI) and cumulative incremental lifetime cancer risk (∑ILCR) values in mango were above one and the moderate risk limit, respectively. Hence, this study revealed that the consumption of mango fruits in the studied areas may pose noncarcinogenic and carcinogenic adverse health effects. Therefore, regular monitoring of potentially toxic elements in fruits is essential to reduce their levels and prevent these adverse health effects.

## Figures and Tables

**Table 1:** FAAS operating conditions for determining potentially toxic elements in fruit samples.

Elements	Wavelength (nm)	Slit width (nm)	Current (mA)	Energy	Detection limit (mgL^−1^)	Linear range (mgL^−1^)	Flame type
Pb	283.2	0.7	2	3.646	0.080	20	
Cr	357.9	0.7	2	3.760	0.040	10	Air acetylene
Cd	228.9	0.7	2	3.342	0.010	2	
Cu	324.8	0.7	2	3.805	0.005	5	

**Table 2:** Mean concentration of potentially toxic elements in selected fruits in Bahir Dar town, Northwest Ethiopia (mean ± SD, *n* = 9).

Types of fruit		Mean concentration of elements (mgkg^−1^, dry weight)			
		Pb	Cd	Cr	Cu
Banana	Mean ± SD	ND	0.130 ± 0.001	0.773 ± 0.010	0.138 ± 0.002
	%RSD	—	0.8	1.3	1.5
Mango	Mean ± SD	0.576 ± 0.009	1.771 ± 0.012	0.728 ± 0.007	2.699 ± 0.009
	%RSD	1.6	0.7	1.0	0.3
Orange	Mean ± SD	ND	0.147 ± 0.001	0.759 ± 0.025	0.138 ± 0.001
	%RSD	—	0.7	3.3	0.7
FAO/WHO limit (mgkg^−1^) [20]		0.3	0.2	2.3	40

ND: not detected; SD: standard deviation; RSD: relative standard deviation.

**Table 3:** Average daily intake (ADI) of potentially toxic elements in various fruits in Bahir Dar town, Northwest Ethiopia.

Type of samples	ADI values for each element (mgday^−1^ kg^−1^ body weight)				
	Pb	Cd	Cr	Cu	Total intake
Banana	BDL	2.14*E* − 04	1.27*E* − 03	2.27*E* − 04	1.71*E* − 03
Mango	9.46*E* − 04	2.91*E* − 03	1.20*E* − 03	4.43*E* − 03	9.49*E* − 03
Orange	BDL	2.42*E* − 04	1.25*E* − 03	2.26*E* − 04	1.71*E* − 03
Total intake	9.46*E* − 04	3.37*E* − 03	3.72*E* − 03	4.88*E* − 03	1.29*E* − 02
RfD (mgkg^−1^) [11]	3.50*E* − 03	1.00*E* − 03	3.00*E* − 03	4.00*E* − 02	
FAO/WHO limits (mgkg^−1^day^−1^) [20]	0.214 mg	0.06 mg	0.2 mg	3 mg	

BDL = below detection limit.

**Table 4:** Incremental lifetime cancer risk (ILCR) and cumulative cancer risk (∑ILCR) of potentially toxic elements from fruit consumption in Bahir Dar town, Northwest Ethiopia.

Type of samples	ILCR value of each potentially toxic element (mgkg^−1^)				
	Pb	Cd	Cr	∑ILCR	US-EPA limits (mgkg^−1^) [11]
Banana	BDL	8.12*E* − 05	6.35*E* − 04	7.16*E* − 04	ILCR < 10^−6^^a^ ILCR < 1 × 10^−4^^b^
Mango	8.04*E* − 06	1.11*E* − 03	5.98*E* − 04	1.71*E* − 03	
Orange	BDL	9.18*E* − 05	6.23*E* − 04	7.15*E* − 04	
∑ILCR	8.04*E* − 06	1.28*E* − 03	1.86*E* − 03		
CSF_o_ [11]	0.0085	0.38	0.5		

^a^US-EPA recommended safe limit (ILCR˂1 × 10^−6^). ^b^Threshold risk limit (ILCR < 1 × 10^−4^).

## Data Availability

All datasets used are included in the article.
